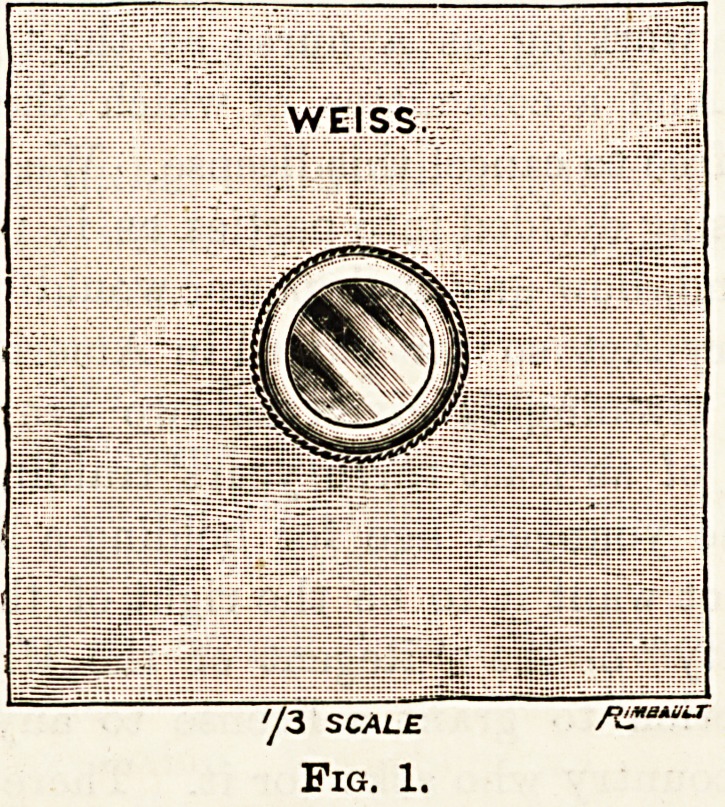# The Hospital. Nursing Section

**Published:** 1903-06-27

**Authors:** 


### The Hospital.

### Nursing Section.

Contributions for this Section of "The Hospital" should be addressed to the Editor, "The Hospital" Nursing Section, 28 & 29 Southampton Street, Strand, London, W.C.

No. 874.—Vol. XXXIV. *SATURDAY, JUNE* 27, 1903.

### Notes on News from the Nursing World.

### THE BISHOP IN NURSING.

We have heard a good deal about the "priest in politics," but the "bishop in nursing" is a novelty. Nor does the incursion of the Roman Catholic Bishop of Ardagh into a region which is not familiar to him prompt us to hope that his example will be followed. In the course of a long letter which the Bishop has written to the guardians of the Granard Union, he says that he insists upon the retirement at once of the nuns from the Union Hospital on the threefold ground of religion, morality, and self-respect. He proceeds to declare that the action of the Irish Local Government Board in this case bodes incalculable evil for our public institutions in the future. He is, of course, entitled to his opinions, and as he conceives it to be his duty as bishop to forbid the nuns to remain at the Granard Hospital, there is nothing more to be said on that point, except that his action is likely to prove an argument against the employment of nuns as nurses anywhere. The letter of "A Catholic Doctor" in another column should be read in this connection. But the Bishop goes on to deliver a tirade against the Local Government Board in which he insinuates that atheistical doctrine is at the bottom of this policy. This only serves to show that in the interests of common sense and charity Irish ecclesiastics had better leave the question of nursing in Poor-law infirmaries to Poor-law officials.

### A CO-OPERATIVE SCHEME FOR INDIA.

Under the heading of "Nurses in India" an appeal is made to the public to find the sum of £5,000 in order to start what is called a Nurses' Co-operation in the Indian Empire. The members of the committee are ladies and gentlemen of high social position, and the Marchioness of Lansdowne, the Marchioness of Ripon, and the Countess of Elgin, wives of ex-Viceroys of India, have given their names as patronesses. The need for good private nurses in India, especially in the North-West Provinces and the Punjab, is urged as a sufficient reason for asking people to provide the sum which, it is calculated, will be required to meet initial expenses, such as passage and outfit for each nurse, the furnishing of the home in India, and the establishment of a reserve fund for emergencies. The committee feel sure that "Anglo-Indians who have themselves realised the great need there is for good nurses in India, and also those whose friends and relatives are now serving there, will come forward to help." There is clearly a special reason why they should, but we do not think that outside their circle much financial support can be expected. A nurses' co-operation is not a charity, but a means of providing employment for a number of nurses who in serving the community benefit themselves. For such an institution it is quite possible that there may be room in India no less than in South Africa, and the Nurses' Co-operation at Johannesburg, which was started since the close of the war, is already a flourishing concern. If not started by nurses themselves the name of co-operation is a misnomer.

### ON QUARANTINE DUTY.

The United States Marine Hospital Service employ nurses for quarantine duty. One of them at present holding that position was previously an army nurse, and she is stationed on Angel Island. Her duties consist in going out with the commanding officer to meet all vessels from the East and making an examination of the women on board for symptoms of bubonic plague or other contagious diseases. Any who have a rise in temperature are at once reported to the medical officer. The most difficult part of the duty at first is learning to climb up the sides of the huge steamers by means of a rope ladder. If a patient off any incoming vessel is found to be suffering from an infectious illness the whole of the crew and passengers are kept in quarantine for a certain time, whilst the vessel, clothing, etc., are fumigated. The passengers have to be accommodated in large buildings, one for cabin passengers, two for ship's officers, and three large barracks for steerage passengers—two for Chinese and one for Japanese. On such occasions the nurse has also to superintend the taking of antiseptic baths. The quarantine station at Angel Island is said to be one of the most completely equipped in the world.

### AN INSULT TO NURSES.

The secretaries of the Eastham Ferry Pleasure Gardens and Hotel Company, Limited, have sent to the secretaries of the hospitals in Liverpool and the neighbourhood a type-written letter to the following effect:—"Our directors are making arrangements for a competition for the nurses of Liverpool and district for a circular walk about five miles from Eastham Gardens. There will be four or five valuable prizes given, followed by a garden party, amusements, and refreshments free to competitors and nurses in uniform. We shall feel obliged if you will kindly bring this before your nurses, and furnish us with any names you may think would be likely to enter, and at the same time inform us the best and most convenient day in the week for your nursing staff. It is the intention to have two competitions, to give all a chance of competing." We entirely agree with the correspondent who encloses us the circular that this proposal is "most insulting and disgraceful." The dates of the competition are not apparently fixed, and it may therefore be hoped that the reception given by the local hospitals to the project will be of such a character as to ensure its abandonment. It is certain that no self-respecting nurse will bring contempt upon her uniform by wearing it through the public streets in order to engage in a "professional walk" for the benefit of the brewery company who own the pleasure gardens at East-ham, even with the prospect of "refreshments free" whether she wins or loses.

### THE NURSING STAFF AT ST. PANCRAS INFIRMARIES.

In the annual report of the Guardians of St. Pancras, it is intimated that the training of probationers at the Infirmary, Dartmouth Park Hill, works very satisfactorily, the only drawback being that, "owing to insufficient numbers," the nurses do not always get their allotted time off duty. As the report says, this, of course, is not conducive to their good health. But surely it ought not to be very difficult to increase the numbers to such an extent that the health of the nurses would be adequately protected. At Cook's Terrace Infirmary, which is also under the jurisdiction of the St. Pancras Board, the arrangements for establishing a training school are nearing completion. The proposal was, it is stated, advocated by Dr. Downes of the Local Government Board, and it lately received the sanction of the Guardians.

### PORTSMOUTH GUARDIANS AND NURSING APPOINTMENTS.

An unjustifiable attack has been made on the members of the infirmary committee and the medical staff of the Portsmouth Workhouse Infirmary by a number of guardians, who nearly succeeded last week in a proposal to alter the present system of the selection of candidates for the position of nurses. Yet, the grounds on which it was sought to effect a change were not only feeble in the extreme, but actually supplied a strong reason for continuing the existing arrangement. The sole pretext for hostile criticism of the infirmary committee and the officials of the Board, was that they refused to appoint as charge nurse a young lady who had been a probationer at the Royal Hospital, but whom neither the late, nor the present, matron of that institution was able to recommend. Therefore, her name was not presented by the officials for election. In the opinion of some of the Portsmouth guardians, the circumstance that the probationer had testimonials "from gentlemen of undisputed reputation," was held to be more important than the views of the experts under whom she had been trained. This will certainly not be the general view of the matter; and as the guardian who introduced the subject said that the matron of the Portsmouth Infirmary is an exceptionally good officer it is curious that he should have made an effort to question a decision for which she was largely responsible, and that an attempt to deprive her of authority should only have been rejected by the casting vote of the chairman. In any case, the adoption of the proposal to invest the Board with responsibility for the choice of individual nurses would have been bad policy.

### AUSTRALASIAN TRAINED NURSES.

From Sydney we have received the first copy of the *Australian Nurses' Journal*, which is henceforth to be issued quarterly as the journal of the Australasian Trained Nurses' Association. The contents consist, mainly, of a *résumé* of the work of the Association, and a lecture by one of the physicians of Prince Alfred Hospital. We notice that the council have decided to register nurses' homes, one of the conditions on which they may be published in the register being that they shall receive only nurses who are on the Register of General Nurses, or on the Register of Midwifery Nurses of the Association. The council congratulate the nurses of New Zealand on the advent of State Registration, but demur to the provision which enacts a three-years' course irrespective of the size of the hospital at which training takes place. The inevitable result of intervention on the part of the State in any country must, however, be a cast-iron system of nursing.

### SUMMER ENTERTAINMENTS AND NURSING INSTITUTIONS.

Several extraordinary efforts on behalf of nursing organisations are reported from the country. At Southport the receipts at a garden fête in the rectory grounds amounted to upwards of £162; at Horwich, near Bolton, a cheque for £120 has been handed to the treasurer of the Nursing Association as the proceeds of the May festival; and at Southend an American Bee has yielded nearly £100. These summer entertainments have not only the primary recommendation of bringing grist to the mill where it is sorely needed, they are also pleasant social functions, at which opportunities are afforded that otherwise might not arise, for discussing the obligation of the community to provide for the nursing of the sick poor. So long as they are not made the excuse for slackening of regular efforts to obtain subscriptions and donations, they cannot do otherwise than benefit the cause both directly and indirectly.

### NURSES' UNION.

The annual meeting of the Nurses' Union and its Medical Missionary Sale of Work was held on Friday last at Morley Hall, Hanover Square. The nurses, on arriving, were received by Miss Dashwood. Although the weather was very unfavourable the sale proved to be a great success, over £20 being taken. There was some excellent music (vocal and instrumental), several recitations, and two missionary meetings. The speakers were Miss Davies (Gorakhpur, India), Rev. Norton Duncan, Dr. Herbert Lankester (C.M.S.), and the Rev. W. H. Griffith Thomas. One of the nurses present has already declared her intention of becoming a medical missionary.

### THE TRAINING OF DISTRICT PROBATIONERS.

The Hastings District Nursing Association having applied to the Hastings Board of Guardians to be allowed to send a probationer to the Workhouse for training, the Guardians have agreed to grant the application on condition that only one probationer is to be sent at a time and that she remains for not less than one month, and preferably for three months. As an inducement to permit the probationer to remain for three months she is, in that event, to have rations free, but if the period is less than three months, there will be a charge of five shillings per week.

### THE AMERICAN GOVERNMENT AND THEIR NURSES.

The quarters provided for the nurses in Manila, Philippine Islands, by the American Government are palatial, the house being formerly the home of a Spanish admiral, and the building is one of the handsomest in the Island. It is a structure in Spanish and Moorish architecture, set in a garden of palms, mango and banana-trees with blooming flowers on all sides. The grounds slope down in the rear to the river's bank, and the nurses have the privilege of using a private stone dock when they want a sail on the Pasig at sunset or on a moonlight night. The doors of the house are very large, and some are of plate glass, a great extravagance in Manila, where glass is so very expensive and so hard to procure, that many windows are made of oyster-shells. There are no carpets on the floors, but they are highly polished, the native boys daily skating over them with coarse cloths dipped in petroleum wrapped round their bare feet. Walls are polished with banana leaves and the same fluid. The knobs and hinges throughout the house are of silver, and there is a roof garden from which a beautiful view is obtainable.

### CARDINAL VAUGHAN'S NURSES.

During the whole of his serious illness Cardinal Vaughan, who died on Friday night, was attended by male nurses. They were supplied by the Temperance Male Nurses' Co-operation, Limited, of Great Marylebone Street, Portland Place, and Manchester. Lately his Eminence required constant attention both night and day.

### THREE MONTHS' LEAVE FOR A DISTRICT NURSE.

At the annual meeting of the Elgin District Nursing Association, the chairman congratulated the members on the manner in which the work had been carried out, and also on the state of the finances. The number of cases received was 276, and the number of visits paid, 5,678. At the monthly meeting in April, it was reported that one of the nurses had broken down in health. The committee, believing that her illness had been aggravated, if not caused, by excessive zeal in the performance of her duties, agreed to give her leave of absence for three months, and pay her salary for that period, engaging a nurse to take her place in the interval. The chairman, in referring to the incident, partly attributed the satisfactory support given to the organisation to the high appreciation of her services in the district. The receipts have increased from £208 to £240, and the balance in hand at the end of the year was £14.

### LEICESTER INFIRMARY NURSES.

Nurses who were trained at the Leicester Infirmary, or have belonged to the staff, will be interested to hear of the formation of a league in connection with that institution. At the first meeting, which was held the other day, honorary officers were elected, Miss G. A. Rogers, the matron, being of course president, a constitution and bye-laws were drawn up, and it was decided to hold an annual general and social meeting in June, to publish a yearly journal in October, and to have a badge. For the benefit of the Leicester Infirmary Nurses' League, two rooms have been taken for this season in a house at Rothby, five miles from the town, to which members can go whenever they like and get "hospital tea" free. A large field has been placed at their disposal by its owner.

### THE CONFERENCE ON WORKHOUSE NURSING.

The important conference on Workhouse Nursing which will be held on Monday afternoon next at 10 Great George Street, Westminster, at the instance of the Workhouse Infirmary Nursing Association, is intended for persons interested in the recommendations of the report of the Departmental Committee on Nursing, and it is hoped will be of a thoroughly representative character.

### STROOD AND FRINDSBURY NURSING ASSOCIATION.

A garden fête in aid of the funds of the Strood and Frindsbury District Nursing Association will be held in the charming grounds of the secretary on Tuesday and Wednesday next. The fourth annual report of the organisation shows that the number of cases visited was 5,574, the new cases nursed during the year being 239. Though the good work of the nurses has been maintained and appreciated, the finances are not so flourishing as they should be, and it is hoped that if the weather be fine the outstanding liabilities of nearly £50 will be defrayed by the receipts at the garden fête.

### NURSES FROM SOUTH AFRICA.

The s.s. *Nubia*, which arrived at Southampton on Monday, had on board Nursing Sister Jacob, of Queen Alexandra's Imperial Military Nursing Service, on three months' leave; and Sisters E. Macrae, E. A. Powner, G. Wood, and H. M. Curtis, of the Army Nursing Reserve Service. Three of these ladies return in consequence of the reduction of the establishment, but Miss Curtis is an invalid on sick leave, suffering from neuritis.

### GOOD PROGRESS AT TROON.

The Troon District Nursing Association, which was founded in 1894, has made excellent progress, and in view of the great increase of work it has been decided to appoint a second nurse. Last year, not including the month when the duties were discharged by a temporary nurse, the number of cases visited was 184, and of visits paid 4,100, as compared with 109 cases and 1,952 visits the year after the Association was established. Thanks largely to the kindness of the Duke of Portland, who has given a site, the committee have decided to build a home for the district nurses at a cost of £1,200, and some substantial donations have been received.

### SOWERBY BRIDGE ASSOCIATION.

Excellent work is being done at Sowerby Bridge, and though the funds at the disposal of the nursing association only just cover its expenses, the small balance is on the right side. The report, which was adopted after a short discussion at the annual meeting, shows that 138 cases were nursed and 2,870 visits paid. The organisation has now been in existence for five years, and the committee claim that the result of the work has been of some economic value and a saving of money to other public bodies.

### The Nursing Outlook.

"From magnanimity, all fear above;
From nobler recompense, above applause,
Which owes to man's short outlook all its charm."

### REGISTRATION OF NURSES.

Princess Christian, who has had probably the fullest experience of the movement of any Englishwoman of eminence, wisely remarked the other day, that the State could not register nurses till there was agreement amongst the hospitals. All who are well informed in nursing matters know that the registration movement in Great Britain is, at present, merely the plaything of outsiders opposed to the principal training schools and large London hospitals, and so possesses little if any interest for practical people. If any substantial good to the public, or the nurses, or both, could be expected to result from a system of registration for nurses it would speedily be adopted in this country. As matters stand at present the blocking of the way is, perhaps, as well, for registration, though it may come some day, will merely stereotype the state of affairs it finds, and at present the diversity in the value of certificates and the inequality in education in the different hospitals is very serious. It is essential that all who aim at the efficiency of British nursing should combine and work first for a more uniform standard of training and teaching.

It is the fashion of those who write and speak most about the registration of nurses to point with scorn to the present state of affairs in England, and to laud the introduction of legislation for the registration of nurses in the United States. Now our American cousins lay down in their Registration Bills, "That nothing shall in any manner whatever curtail or abridge the right and privilege of any person to pursue the vocation of a nurse, whether trained or untrained, registered or not registered" (Carolina). This clause clearly reveals the spirit in which such legislation is enacted in the United States. An agitation is got up to secure an Act on a particular matter, which is duly passed but never enforced, for the simple reason, that no machinery for its enforcement is set up or probably desired. There is no utility in an Act of the legislature unless that Act is so drawn as to clearly define the purposes aimed at, and to secure the provision of funds and an adequate and properly constituted authority to ensure its enforcement when passed. None of the American Registration Bills fulfil these essential conditions, and for all practical purposes they might just as well have no existence. And here we think it well to warn nurses against writers and speakers who, when advocating the registration of nurses in England, quote the action of the State legislatures in America as pointing the way, and as proving that the British nursing authorities are all behind the times and asleep. The truth is, that in this country we are accustomed to move slowly, because we recognise the value of time, and know full well that to secure efficiency we must be practical. Now, for practical purposes, the registration of nurses in this country to-day could fulfil no useful purpose, for the simple reason, that any scheme of registration must accept the condition of affairs in the nursing world as it exists at the time such a scheme is commenced. But so much remains to be done in the way of codification and consolidation of reforms in regard to the training and certification of nurses in England, that no scheme of registration for nurses can have any real interest for practical politicians.

Again, the American legislation only gives what the Royal British Nurses' Association gives in England by Royal Charter, *i e.*, the right to its members to put certain initials after their names, and hold a "license" on paying for it. In America, too, the only qualifications for the license are to be over twenty-one years of age and to be working as a nurse at the time the Act passes. And a committee, of which the quorum is three, is free to cancel this license. We can imagine, with nursing divided into parties as it is at present in England, how any such power would be abused!

In every Act so far passed in America the ideal length of training is given as two years; we want an ideal of at least three years' training and with 25 as the youngest age for holding a license. We also do not want it to be the right of the "Clerk of the County" or the "Regent of the University" at his discretion to grant a license to any nurse from another country who asks for it. There is no better motto than *festina lentè*—hasten slowly. The first step in England should be for the matrons and teachers of the great metropolitan hospitals to accept the Princess Christian's suggestion, and meet and confer on the possibility of agreeing to an ideal curriculum. This would do much to raise the standard of training and help the different hospital boards to know at what they ought to aim. With cottage hospitals of 20 beds giving certificates at the end of one year, and asking a premium from their probationers; with large hospitals extending their training to four and five years, and often training all individuality out of their nurses; with nursing homes also posing as training schools; and with large numbers of good private nurses now at work who hold no certificate whatever; we feel that the profession is in a state so chaotic that the immediate necessity is to try and get a pronouncement from such nursing leaders as the matrons and lecturers of St. Thomas's, the London, Guy's, King's, and Westminster. So far as we know, these authorities have not yet spoken on the present nursing crisis; we await their utterance a[illegible] the one authoritative declaration that can result [illegible] practical progress.

### Lectures on Ophthalmic Nursing.

By A. S. Cobbledick, M.D., B.S.Lond., Senior Clinical Assistant Royal Eye Hospital, late House-Surgeon and Registrar, Royal Eye Hospital.

LECTURE XIII.—TREATMENT OF PURULENT CONJUNCTIVITIS (*continued from page* 143).—PHLYCTENULAR OPHTHALMIA.

There are a few more points of importance in the treatment of this disease which were not included in the last lecture. I should like to again impress on nurses the importance of taking every precaution against infection, not only by protecting the eyes with large circular glasses, but by cleansing the hands carefully in an antiseptic lotion immediately after attending to a case.

It is also most important to explain to the patient or the baby's relatives the contagiousness of the disease; that the patient must be isolated and have his or her own towel and flannel. When one eye only is affected, every care must be taken to prevent infection of the sound eye. The patient should lie on the same side as the eye affected, so as to prevent any discharge passing over the bridge of the nose into the sound eye. It is better, however, in order to completely diminish any risk of infection, to keep the sound eye isolated by covering it with a pad of wool and gauze, the

Fig. 1.

edges of which must be firmly sealed by means of flexile collodion. A more comfortable method is to apply a Buller's shield (fig. 1).

Such a shield can be improvised by means of an ordinary watch glass and some strong adhesive plaster. Much care must be taken to make the edge of the plaster fit the bridge of the nose, and, if necessary, flexile collodion should be used in addition. The great advantages of this shield are that it is comfortable and that the patient is able to use the eye.

*Phlyctenular Ophthalmia.*—This term includes phlyctenular conjunctivitis and P. Keratitis. This is one of the most common eye affections in young children; it is seldom found in babies; it very commonly follows an attack of measles or one of the specific fevers. As a rule the children are ill-nourished and miserable specimens of humanity showing signs of a strumous taint, viz.: large swollen and everted lips, glandular enlargements, a depressed bridge of the nose, and not unfrequently they are *mouth breathers*, and suffer from some of the consequences of that condition, viz.: discharge from the ears, tendency to recurrent attacks of bronchitis with chest deformity, loss of appetite, and consequently of flesh; hence, with good reason, this affection has been called *strumous ophthalmia.* Occasionally, however, the child affected is apparently healthy and well nourished.

*Symptoms.*—Mild cases of this disease are confined to the conjunctiva and do not give rise to severe or troublesome symptoms. A yellowish raised spot, called the *phlyctenule*, appears beneath the conjunctiva in one or more places at a variable distance from the corneal margin; the conjunctival vessels immediately around the phlyctenule become engorged and produce an area of redness. There is always some attendant pain or discomfort and a slight discharge causes the lids to stick in the morning.

With appropriate treatment these slight cases can be cured in ten days or a fortnight. In the more severe cases the cornea also is invaded with phlyctenules, or the cornea *alone* may be affected. When a phlyctenule has formed in the cornea it breaks down, discharges, and leaves an ulcer of varying size and depth; as the cornea is very abundantly supplied by nerve endings, the ulcer irritates them, causes pain and inability to open the lids or face any but a faint light. The pain and photophobia are the most distressing symptoms, and are frequently accompanied by *spasm* of the lid muscles, so that any attempt to open the lids with the fingers immediately causes their eversion. The spasm is due to a small fissure at the outer canthus, *i.e.*, at the junction of the upper and lower lids externally, which is extremely painful, and bleeds readily on the slightest movement of the lids. This spasm may be so great as to cause undue pressure on the eyeball and hinder healing, or it may continue after the healing of the ulcer is well established.

*The Ulcer.*—The type varies greatly. Small superficial ones near the margin of the cornea are not troublesome. Deep ulcers in the substance of the cornea are always dangerous, firstly, on account of the possibility of perforation, and secondly, on account of the subsequent impairment of vision especially if they are situated near the centre. Sometimes the ulcer spreads from the margin of the cornea towards the centre, healing near the margin, but always supplied by a leash of blood-vessels.

In all cases where the cornea is affected, the conjunctival vessels, leading to the ulcer or ulcers, are enlarged.

*Prognosis.*—Slight cases, limited to the conjunctiva, make a complete recovery in a week or two, but it must be remembered that there is a tendency to recurrence. Superficial corneal phlyctenules, although painful, usually heal readily, and do not necessarily leave any opacity.

The deep ulcers, following large phlyctenules in the substance of the cornea, are often most tedious, and relapses are frequent. All these cases sooner or later, as the patient nears adult life, get well, but frequently with the most disastrous results to vision. The deeper the ulcer the more opaque is the resulting scar, and the nearer the opacity is to the centre of the cornea the greater is the impairment of vision.

*Diagnosis.*—This is seldom difficult. The general appearance of the child, with the accompanying strong aversion to the light, is sufficient on which to base a diagnosis, and also to infer that the cornea is affected. The difficulty is to get a good view of the cornea and determine the extent of the mischief present. As a preliminary, tempt the child to open the eyes in a subdued light; the child can often do this if the corneal affection is not severe, or when it is nearly healed. Manipulation of the lids with the fingers usually increases the spasm so that the lids become everted and the cornea hidden. When this is the case, do not make repeated attempts to retract the lids with the fingers, for there may be a deep ulcer present on the point of rupture.

With lid retractors, the cornea can always be thoroughly examined without any danger of exerting undue pressure on the eyeball. Note the type of ulcer whether deep or shallow, whether placed centrally or peripherally, their number and the stage of breaking down or healing they have reached.

The only corneal troubles with which this condition could be confused are:—

1. The small marginal infiltrations found in purulent conjunctivitis.

2. Interstitial keratitis. In these cases the surface of the corneal is never broken, and nothing like an ulcer forms; the trouble is in the substance of the cornea, and from the commencement it is somewhat opaque and loses its lustre.

3. It might be confused with a condition of the cornea called pannus, caused by the irritation of granules on the inner surface of the upper lid.

Examination of the upper lid in doubtful cases would soon make the diagnosis clear.

### The Higher Training of Midwives.

### MISS GREGORY'S SCHEME.

A well-attended drawing-room meeting was held on Thursday last week at 3 Grosvenor Place by permission of Lady Esther Smith, to discuss a scheme drawn up by Miss Alice Gregory for the higher training of midwives.

The scheme, as set forth in a little pamphlet which was placed in the hands of the meeting, proposes that a general hospital with a maternity annexe should be started in some neighbourhood which is already in need of such an institution; that this hospital, in addition to its primary object of nursing the sick poor, should be recognised as a National Training School for Midwives, and that within its walls educated women should receive an 18 months' course of general and monthly nursing, prior to a six months' course of district midwifery—a two years' training in all—after which they should be drafted out into country districts. It is also proposed that the midwives should be supervised periodically from the central organisation, and should return every third year for re-examination and instruction in the advances of modern obstetrics.

### Miss Florence Nightingale's Sympathy.

The chair was taken by the Dean of St. Paul's, who had to communicate to the meeting the disappointing information that Mrs. Scharlieb, M.D., who had promised to speak, had telegraphed her inability to attend on account of an important operation. The Dean also read a telegram from Miss Florence Nightingale, expressing her warm sympathy with the object of the meeting. Miss Nightingale has permitted her name to be added to the list of patrons in token of her cordial approval.

### Educated Women as Midwives.

The Bishop of Stepney reminded his hearers that our national supremacy rested upon the physical well-being of the people. It was a terribly significant fact that in spite of all the advances made in surgery and medicine there had been no decrease in the death-rate of infants in the last fifty years. In 1901, fifteen out of every hundred children who were to have replenished the population died before their first birthday, owing to neglect at the time of their birth, not to speak of those who were maimed for life and grew up to be a burden to society. Besides this infant mortality numbers of mothers died from neglect and carelessness. From one-fourth to one-third of the inmates of blind asylums were there because of the neglect of proper washing at the time of birth. The practice of midwifery had been left far too long in the hands of women who were ignorant of all the most elementary rules of nursing, and were often governed by mediæval superstitions. They must now consider on what lines the reform in the system should be based. Two schemes were laid before the public: The first and simplest plan was to give the present class of nurses as much training as would prevent them from making any serious mistakes in the treatment of their cases. The second plan, as suggested by Miss Alice Gregory, was to raise the class of nurses and the training they were to receive. The Bishop, speaking from his own experience, said that educated women who gave themselves up to the work showed great sympathy with the poor and performed their work with much tact and devotion. The difficulty that confronted them was to obtain a sufficient number of educated women to take up the work. He earnestly hoped that Miss Gregory's plan would be adopted.

### A Hospital for Woolwich.

Sir Edmund Hay Currie said that Miss Gregory's scheme must be the right course for it was the object of all the hospitals nowadays to get the best class of women and give them the best kind of training. Miss Gregory was anxious to start a hospital in South London with an annexe for maternity cases. It was a great scandal that there were 750,000 in South London, a greater population than Liverpool, without any hospital accommodation whatever. Woolwich had no hospital and a population of nearly 120,000. There could not be the slightest doubt that it was therefore the place where the hospital should be started. It was absolutely necessary that something should be done in South London and a hospital to contain 70 beds established.

### To Popularise Midwifery.

Miss Gregory, in explaining her proposals, said that what was required of midwives was fair mental capacity, technical skill, and practical knowledge of nursing; they must also know when to send for the doctor and how to prepare for the operation. If they could get the three-years' trained nurses to take up this work they could desire nothing better, but this they found they could not do. The matron of one of the largest London hospitals said that practically none of her nurses would take it up. The profession of midwifery had fallen into such disrepute that fully-trained nurses had a great contempt for it. In order, therefore, to popularise it amongst educated women, it was necessary to establish a special training school, belonging entirely to themselves, where they should learn midwifery and monthly training. Now was the great opportunity for them to decide what ideal they were going to set before them. Miss Gregory then drew attention to the training received by midwives in other European countries, and pointed out that England was far behind in this respect. In France no midwife was allowed to practise without having received two years' training. In Holland, in addition to two years' training, the nurses had to pass examinations, and if they failed they had to prolong their course to three and four years till successful. In Russia three years' training was required. After describing some of her experiences in Somersetshire, where she had been acting as midwife for some years, Miss Gregory expressed her opinion that a fee of 8s. could in all cases be obtained; this was what she herself had charged and in all her experience had only lost one fee. They hoped to be able to get support from the various county councils in the shape of scholarships for pupils in general hospitals.

Dr. Annie McCall, director of the Clapham Maternity

Hospital, said that one of the advantages of having educated women as midwives would be the spread of popular hygiene in the cottages. Dr. McCall said she was confident that if Miss Gregory were given a chance of starting her scheme it would work successfully.

A Conference of Experts.

The Chairman then announced that Mr. Asquith, whom they had hoped to listen to, had been prevented from coming. He added that Miss Gregory intended to call together a general conference of experts in the autumn or winter to consider the best means of carrying the scheme into execution under the auspices of Princess Christian, who had expressed great sympathy with the movement.

Dr. Cullingworth, member of the Central Midwives Board, said that on behalf of the Board he welcomed this and all other efforts to improve the training of midwives and to raise their status. The two most admirable points in the scheme were, first, the ensuring a good training in general nursing, and, second, prolonging the special training in midwifery.

A vote of thanks to Lady Esther Smith was proposed by Sir Sydney Waterlow, who expressed himself in hearty concurrence with Miss Gregory's plan.

### The Nurses' Missionary Union.

THE inaugural meeting of the Nurses' Missionary Union was held in the Church Room adjoining Holy Trinity Church, Hampstead, on Wednesday in last week, when a number of nurses were present. The chair was taken by Mr. H. T. Hodgkin, M.B, of St. Thomas's Hospital, and the speeches were arranged in two sections, so that those who could not stay during the whole time were able to gain a clear idea of the aims of the society.

The Needs of China.

Dr. J. Preston Maxwell spoke of the urgent need for trained nurses in China. A doctor, he said, did not realise until he went abroad how much was done for him by the nurse; he had frequently not only to prepare everything before an operation, but also to act as nurse afterwards. Nursing, properly so-called, did not exist in China except in one or two specially-provided hospitals. It was done by the doctor, by native students, who, if conscientious, made good nurses, by men and women hired for the purpose, and by the friends of the patients. The last-named were a source of great trial; they assisted the patient to elude the vigilance of the doctor, introduced forbidden food, cooking it on the premises, and they sometimes removed the patient in the night. The hired attendants were not much better, and he had known one case in which a woman did nothing for the patient, but set the entire ward to do some needlework for herself. In times of pressure, patient and "nurse" occupied the same bed. He had known the patients of one ward engage a caterer from the city, plan a midnight feast, and invite patients from other wards to join them. Such an act of insubordination would, of course, be impossible if there were a properly trained nursing staff, and this was sadly needed in China, not only for the patients' sake, but as an educational force. In illustration of the need, Dr. Maxwell told how a man died of confluent small-pox. Under the bed were the pigs, all round were the fowls, the buffalo used for ploughing was at the door, and the bed was shared by the patient's brother.

Dr. Maxwell urged that one of the first requisites for a nurse going to China was adaptability; there were no English beds with sheets and blankets; the beds were of wood, and the patient rolled himself in a quilt. Baths were unknown, and at the mere suggestion the patient would probably leave the hospital. The chief requisite, however, was Christian zeal, and he wished to impress upon the nurses the fact that the call to take up work in mission hospitals was really a call to missionary work. He deprecated what he saw to be a tendency of modern times to look upon medical work as a form of philanthropy, rather than as a means of spreading the Gospel. What China needed was an efficient nursing body, and every mission hospital ought to have at least one or two nurses. They must be highly qualified, and midwifery should form part of their training. Alluding to the training of native women, he said that this must be done by nurses; it was a branch of medical science that must be developed independently of the doctor, who had neither the time nor the requisite knowledge of modern nursing developments to undertake it. It must not be looked upon as simply an adjunct of the hospital. The matter was one that was bound to come prominently into public notice, and he advocated the claims of the Union, first because a missionary spirit should form part of the Christian life, secondly because the need was so great, and thirdly because practically every country in the world was now open to missionary enterprise.

The aims of the Union were then explained by Miss K. Miller, who said that it had been called into existence for the purpose of extending the knowledge of missionary work among nurses, and of helping to meet the demand for their services abroad. It was hoped by this means to reach every nurse in the hospitals; and wherever facilities were granted, branches would be formed, a secretary appointed, and meetings held for Bible and missionary study. Although an outside organisation, the co-operation of the nurses was necessary to success. Individuals might join the central society if no branch existed in the hospital to which they were attached. It would thus offer a means of preparation for nurses anxious to offer themselves for work in the mission field, as well as forming a link between those who had this end in view.

At the evening meeting an interesting account of her Mission Hospital in Persia was given by Miss Emmeline Stuart, M.D. A provisional constitution was adopted, and a committee, three being nurses, was elected for one year. The motto adopted was, "The evangelisation of the world in this generation."

### TRAVEL NOTES AND QUERIES.

Cheap Quarters in Paris (M. J.)—We do not answer by post unless 2s. 6d. is sent for our Convalescent Fund. You must be cautious in selecting cheap places in Paris. Here are a few addresses—Mme. Ledoux, 20 Rue Clairant Avenue de Clichy, from 6 francs per day. Miss Bromhead, 47 Boulevard Gouviou, St. Cyr, from 30s. per week. Mme. Mansfield, 157 Faubourg St. Honoré, about the same. Mme. Cliché, 1 Villa de la Réunion, Auteuil, 8 francs per day. This last very agreeable. The cheaper places in Paris are constantly moving and closing, so write soon.

Knokke in Belgium (Reta).—Fare, second class return, 9s., *viâ* Tower Bridge and Ostend. Fare beyond Ostend approximately 1s. 4d. Hôtel de Bruges or Hôtel de la Marine. I know of no other place so cheap—the next would be St. Servan in the Bay of St. Malo, but that comes to 7 francs (5s. 10d.) per day. Address Miss Humfrey, 20 Place Constantine, St. Servan, France, or Madame Pallot, Maison Mathias, St. Servan. If you like Cornwall I can recommend Miss Snell, Ivy Cottage, Caerhays, near St. Austell, terms from 18s. to 25s. per week. Tourist ticket from London to St. Austell 26s., carriage on 7s.

Lodgings in Paris (For five years a Subscriber).—Lodgings, as we understand the word, do not exist in Paris. The best I can do for you is the same as for M. J. See above. I can add one other address, Miss Wilson, 3 Rue Pelouze Parc Monceau. You will see that at Mme. Ledoux's you can board for 6 francs per day if for a week or more; thus your expenses each would come to £1 6s., without tips, per week. Unless you are experienced travellers, it would be impossible and unsafe for you to attempt taking rooms and managing for yourselves. No charge for answer thus.

Travel Editor.

### Everybody's Opinion.

### NUNS AS NURSES.

"A Catholic Nurse" writes: I have read with great interest the article which appeared in The Hospital on "Nuns as Nurses," and would like to say that a "religious" criticism on their work is quite outside the province of a secular person. The Sisters of Charity, the little Company of Mary, and the "Sœurs de la Miséricorde" (the three chief nursing orders in England and France) need no reform, and their work is really done from the highest motives—charity and the love of Christ. The life of the "nun" nurse must be the more perfect life than that of the professional nurse, for after all, "God's things are best." I speak from personal experience, and in justice to all religious orders, who number among their ranks the noblest, truest, self-sacrificing women that the world contains.

"A Catholic Doctor" writes: It is useless for nuns to take up nursing unless they do it in the modern scientific manner, and, at least, as well as lay nurses. Otherwise, neither public bodies nor the medical profession will have them. In Ireland the introduction of the nuns into the workhouses was a great improvement upon the old system; but now that the nursing profession usually demands three years' training, with experience, tuition, lectures, examinations, and the study of recent text-books, it behoves our nuns to look to their training or they will fall behind. This is a matter which concerns the head of the Orders and the authorities of the Church. If the nursing nuns are to hold their own and to prove themselves worthy of the splendid reputation which Catholic sisterhoods have gained in so many posts of danger and under the most trying conditions, they must keep well up to the ever-improving standard of the hospital nurse.

### THE NURSES' DINNER-TABLE.

"Superintendent Nurse" writes: In reply to the article entitled "The Nurses' Dinner-table," I should like to say a few words. My experience in a large provincial hospital was that the food was excellent and varied and the cost wonderfully little. Anyone who grumbled there should have been turned out. In my present post as superintendent nurse in a workhouse infirmary I find there is every excuse for grumbling, both as regards quality and monotony, and it seems very difficult to effect much-needed reform. The guardians have been approached on more than one occasion, but it seems very difficult for them to act in the matter. They seem prone to wait till the "more convenient season." My own opinion is that the ration system is an entirely wrong one, and that the superintendent nurse should be given a much freer hand in the nurses' dietary, and if she is at all a capable housekeeper she will be able to give them much greater variety at no greater expenditure. The same feeling exists at other workhouse infirmaries, with the result that a great deal of the nurses' hard-earned money goes to buy food, and nurses as a class are considered improvident. The reason is not far to seek. If not properly fed how can they remain physically fit to attend to their arduous labours, where they are exposed to diseases of all kinds and have very monotonous work. The amount allowed for feeding them seems ample if properly expended, and ought to secure both variety and good quality.

### THE RURAL MIDWIVES' ASSOCIATION.

"Mrs. Heywood Johnstone," Chairman of the Rural Midwives' Association, writes: The allusion of the Bishop of Stepney, in his speech at the meeting in support of Miss Gregory's scheme to-day, to such work as ours, left the impression that our efforts were to take the present untrained midwife, give her a short smattering of midwifery, and then leave her to go on much as before with the dangerous addition of "a little knowledge that is worse than none," as opposed to the better way of a course of two years' careful training. It is quite true that we propose, where possible, to take a respectable conscientious woman who has followed the calling of a midwife as a profession, and (although she may at present claim a certificate if she has been a year at work) to persuade her to take, not a smattering, but a recognised course of training; but we also, and that much more frequently, desire to assist those to follow this profession who till now have not taken it up, and I wish most emphatically to point out that the training we give in midwifery is as long and as thorough as that given by Miss Gregory herself, namely, if necessary, six months. There is a cry that we would send to the poor these imperfectly trained women whom we would not accept for our own class. This, again, is incorrect. The maternity nurses and midwives trained at Queen Charlotte's and other hospitals, who have for years furnished the majority of ladies' nurses, have had a training no longer than that which we are supplying for village midwives. At the same time it follows reason that as they may be called upon to work in places where there are few or next to no appliances (as in one instance when a beer-can was the only available "plant" at hand), it behoves us to teach these women to do without, rather than with, the 50 accessories of a perfectly organised maternity ward, and this can only be taught in the district training of our thickly populated towns, though it would certainly be advisable for them to receive some portion of their training in a maternity hospital when possible. It is not in the midwifery training our lines diverge, but in the 18 months' general training that Miss Gregory would give in the hospital she purposes building. Our women, as a rule, have a short training in this respect—the proof of the pudding is in the eating—and I am not exaggerating when I say thousands have found this *is* sufficient to make a useful village midwife, who, under the doctor, is able to add that of nursing in emergencies that occur in connection with her calling. I think the advocates of the long training sometimes forget that we are within reach of the general practitioner, to whom the patient must be handed over when the ordinary functions of nature take an abnormal form. Year after year have my nurses been able to take such cases under my doctor's direction, and never once has any catastrophe occurred through lack of knowledge. The responsibility has been his, the intelligent obedience theirs. Now under the new order of the Midwives Act will be added the further knowledge of when to call him in. I write this in no spirit of opposition. There is room for Miss Gregory's high ideal—may she go on and prosper!—but her advocates will not strengthen her cause by mistaking or misinterpreting the good and thorough work of those who are stepping into the gap, and—by the utilisation of the known and approved existing training institutions—are sending out the homely woman (forgive the much-condemned adjective) who will have received—not a smattering—but a thorough training in accordance with the rules of the Midwives Board. I think we must all trust the Board to know what is necessary.

### The Nurses' Bookshelf.

A Complete System of Nursing. Written by Medical Men and Nurses. Edited by Honnor Morten, L.O.S. Diploma, Hygiene Certificate, Bedford College, London; formerly Lecturer under the London County Council, etc. Author of "The Nurses' Dictionary," "How to Become a Nurse," etc. (London: Sampson Low, Marston and Co. 1903. Price 7s. 6d. net.)

The "Complete System of Nursing" which Miss Morten has compiled possesses the merits and defects of being the work of many hands. Some of the writers keep strictly to points of nursing, some write as if the book were intended for medical men, and some realise that a book like this will fall into the hands of many untrained persons, who will look to it for guidance in emergencies. The practical value of the chapters thus varies greatly. The writer of the first chapter, on "The Hygiene of the Sick-room," says frankly that, "in private nursing the general arrangements of a sick-room are (as a rule) compromise throughout," and wisely bases her advice on this assumption. On the other hand, the author of the section on kidney diseases addresses his remarks on urine-testing to a nurse in hospital with a whole ward to look after. On the whole we think that the editor has been too considerate of the feelings of her contributors. Thus, she puts some remarks on the treatment of lupus by the Finsen light in her preface, but there is no mention of it in the article on lupus, where one would naturally expect to find it, nor is there in the book any advice as to nursing of cases thus treated. There is no mention of nasal feeding in typhoid cases, although when unconsciousness is present this is a valuable way of administering nourishment. It is mentioned in the treatment of diphtheria, with the mere remark that it "presents little difficulty." Though easier than forced feeding by the œsophagus, with which it is there compared, it is worthy a word of explanation. On the other hand, the chapters on surgical nursing, gynæcology, the nursing of nervous and of mental diseases are admirable, and the chapter on the nursing of ophthalmic affections will be found of the greatest help to the many nurses who have to deal with such cases without having received any special training in it. That on drugs and their administration is also very valuable. In short, it may be said that this book will be of the greatest use to a nurse who has received a sound general training, but has not devoted herself to any speciality. It is not meant for the amateur nurse—for whom more than enough literature is already provided—but it should be very useful to trained nurses in the many emergencies with which they may be called upon to deal, while its convenient size makes it a volume which can be carried from place to place without inconvenience, even by one who wishes to limit her impedimenta to the utmost.

### Novelties for Nurses.

By Our Shopping Correspondent.

### THE BEST EAU DE COLOGNE AND A NEW SCENT.

Whilst the manufacturers of the celebrated 4711 Eau de Cologne fully maintain for it the reputation which it has now gained as the best eau de Cologne, they do not forget that in respect to other perfumes novelty is a great attraction. The latest perfume they have introduced is a delicious extract of violets, called Violetta Graziella. It is daintily prepared in pretty bottles with stopper, on which is engraved a violet, and tied with violet and white ribbons, so that it forms an attractive little gift for the toilette table. The Eau de Cologne and Violetta Graziella can be obtained where perfumes are sold, and at the depôt at 162 New Bond Street, London, W.

### THE NURSES' CO-OPERATIVE DRESSMAKING ASSOCIATION.

I have received some nice patterns of materials for cloaks and dresses from the Nurses' Co-operative Dressmaking and Outfitting Association, 82 Wellington Road South, Stockport. The "Danco" serge, which is a speciality of this firm, is in black and navy blue; the price is from 1s. 5¾d. per yard, and it is 50 inches wide. The firm undertakes to make dresses from £1 4s. 6d. Measurements must of course be sent with the order, and it is claimed that the system is so simple that mistakes are impossible. The dresses are lined, and may be had in Estamene serge, navy and black; alpaca, beige, cloth, etc., as well as in the material mentioned above. Cotton dresses are made from 12s. 6d. in art linens, galateas, plain and striped, zephyrs, drills, regattas, etc. The price of nurses' cloaks is from 22s., and the pattern of Danco Cravenette which has been sent me is a nice fine serge which it is said will neither change colour, shrink when wetted or washed, cockle in rain, nor suffer from exposure to sun, sea-water or sea air. The patterns of gauze for veils are charmingly soft and silky in texture; the price is 1s. 11¾d. per yard 26 inches wide. The bonnets supplied by this firm are made of fine pedal straw, and may be had in the Marie Stuart shape, trimmed with silk ribbon velvet. The price is 7s. 11½d., 2s. 6d. extra with veil. Collars and cuffs, gloves, nurses' wallets, scissors, and other accessories are also supplied, and a full catalogue will be forwarded on application. This Association has recently opened a new department for the convenience of tired and busy nurses who need garments of any description repaired. The terms are said to be exceedingly moderate, and are arranged according to the work required. "The Nurses' Mendery" should be a popular branch of the firm's at Stockport.

### Appointments.

**[No charge is made for announcements under this head, and we are always glad to receive, and publish, appointments. But it is essential that in all cases the school of training should be given.]**

Grimsby and District Hospital.—Miss Ruth Des Forges has been appointed night sister. She was trained at the Seamen's Hospital, Greenwich, and the Women's Hospital, Soho Square, London. She has since been nurse at Hull General Infirmary, and sister at Ramsgate Hospital. Miss Frances Crichton, the new matron, was night superintendent and home sister, not assistant matron, at the General Hospital, Birmingham.

Lincoln Workhouse Infirmary.—Miss Hilda Lea has been appointed superintendent nurse. She was trained at Brownlow Hill Infirmary, Liverpool, and has since been attached to the private staff of the Royal Berks Hospital at Reading.

Lodge Moor Hospital, Sheffield.—Miss Catherine Marie Duffy has been appointed assistant matron. She was trained at the Royal Albert Edward Infirmary, Wigan, and had since held the position of sister at Park Hill Hospital, Liverpool, and night superintendent at Lodge Moor Hospital, Sheffield.

Radcliffe, Ramsbottom, Whitefield, and Bury Isolation Hospital.—Miss Nellie Sinclair has been appointed charge nurse. She was trained at the General Hospital, Calcutta, and has since been charge nurse at the Isolation Camp for Small-pox, Edmonton, N., and charge night nurse at Enfield Cottage Hospital.

Union Infirmary, Dudley.—Miss Elizabeth Wilson has been appointed superintendent nurse. She was trained at St. George's Infirmary, Fulham, and has joined the Meath Workhouse Nursing Association.

Union Infirmary, Plymouth.—Miss Alice Collett has been appointed assistant nurse. She was trained by the Meath Workhouse Nursing Association at The Home for Invalids, Aubert Park, Highbury.

Victoria Hospital, Blackpool.—Miss Hannah Mellor has been appointed sister. She was trained at the Royal Infirmary, Sheffield, and held the post of staff nurse for 12 months. She has since been attached to the Nice Nursing Institute.

Wheatenhurst Union.—Miss Nellie Pitt has been appointed assistant nurse. She was trained by the Meath Workhouse Nursing Association at St. Peter's Home, Maybury Hill, Woking.

### "The Hospital" Convalescent Fund.

The Hon. Sec. begs to acknowledge with thanks the receipt of two contributions of 2s. 6d. each per the Travel Editor of The Hospital.

### Echoes from the Outside World.

### Movements of Royalty.

The King and Queen returned to London from Windsor Castle on Monday, and the Queen with her suite attended the performance of "Otello" at the Royal Opera in the evening. On Tuesday they gave a children's party at the palace in honour of the birthday of the King's eldest grandson, Prince Edward of Wales, who has now reached the age of nine. About 150 young guests were invited. The King went over to Marlborough House early to give his congratulations in person, and later the little Prince with two of his brothers and his sister drove to the palace for his birthday festivities. During the afternoon the King was present at the Chapel Royal St. James's, and acted as sponsor to the infant son of the Earl and Countess of Lytton. On Wednesday his Majesty went to Park Royal for the Agricultural Show, and Friday is the day appointed for the King's birthday to be observed.

### The Servian Revolution.

In the House of Lords on Friday the Foreign Secretary announced that his Majesty's Government had come to the conclusion that our representative in Servia had better not remain in Belgrade when the new *régime* was inaugurated, and accordingly Sir George Bonham, who has hitherto acted as British Minister, left the Servian capital on Tuesday for London. The Servian Parliamentary deputations to King Peter have declared that the demands of Russia for the punishment of the murderers of the King and Queen are incapable of realisation, and the *Mala* affirms that "the act which has been committed in the Servian capital has saved the honour of Servia, has exalted her prestige, and has restored peace and tranquility to the country." King Peter arrived in Belgrade on Wednesday morning.

### The late Cardinal Vaughan.

The death of Cardinal Vaughan, the head of the Roman Catholic Church in England, took place on Friday night last at St. Joseph's Missionary College, Mill Hill. It was not altogether unexpected, for his Eminence had been seriously ill since last February, and a temporary improvement did not justify hopes of recovery. Cardinal Vaughan, who was in his seventy-second year, was the eldest son of the late Lieut.-Colonel Vaughan of Courtfield, Monmouthshire. Originally he intended to enter the Army, but changing his mind, was ordained a priest at Lucca in October, 1855. In his early days he founded the first missionary college of his church in this country at Mill Hill, carrying on his work from hand to mouth, but always obtaining financial help when it was most needed. In October, 1872, he was consecrated Bishop of Salford, and worked in Lancashire for twenty years with great energy. On the death of Cardinal Manning he was nominated Archbishop of Westminster, and on January 16th was created Cardinal Priest of the title of St. Gregory on the Cœlian Hill. Cardinal Vaughan had a handsome and stately presence, was a clear and forcible speaker, and was in all respects a devoted ecclesiastic. Tributes of sympathy to the members of his Church in the loss they have sustained have been forthcoming from all quarters.

### The Proposed Abolition of Barmaids

An attempt to bring about the compulsory abolition of barmaids was made last week in the discussion in the Grand Committee on Trade of the Scotch Licensing Bill. Mr. Crombie proposed that the licensing authority should have power to make a bye-law preventing employment of barmaids, and he got fourteen members of the House of Commons to vote with him. On the other hand, the Lord Advocate persuaded nineteen to vote against it, and in the course of his speech he said that a regulation of the kind suggested was very close to the ridiculous and was an interference both with people's ordinary discretion and with honest employment for women. It was all very well to contend that a woman encountered temptations by going to a liquor shop; but he maintained that she had a good deal more temptation if she were left starving outside a liquor shop.

### The Moat Farm Murder.

On Tuesday, at Chelmsford Assize Court, Samuel Herbert Dougal was sentenced to death for the murder of Miss Camille Holland, a lady with whom he lived at Moat Farm, Clavering, Essex. The court on the last day of the trial was exceedingly crowded, ladies especially exhibiting the utmost eagerness to obtain seats. The prisoner appeared quite at his ease all the time, and nodded to a local Essex farmer who was present. He took much interest in the evidence tendered by the old man who filled up the ditch where Miss Holland's body was afterwards found, by the bootmaker who made the tiny boots found on Miss Holland's feet, and by the cashier of the Birkbeck Bank, who stated that Dougal opened an account there on October 14th, 1899, five months after Miss Holland had disappeared, paying in a cheque of £670, represented by a cheque drawn apparently by Miss Holland on her own bank. From that time large sums of money were transferred from Miss Holland's to Dougal's account. Dougal was arrested at the Bank of England for forgery on March of this year, the charge of murder being added subsequently. Counsel for the defence laid stress on the fact that none of the clothing found on the dead body had Miss Holland's initials upon it, though it was proved that all her clothing was so marked. But Mr. Justice Wright, in summing up, said that the jury must ask themselves if this was not the body of Miss Holland, where was the body of Miss Holland, and commented on the fact that Dougal never took any steps to find out where Miss Holland had disappeared to after, as he stated, driving her to the station on the day she left Moat Farm. The jury retired at five minutes to four, and returned within the hour.

### Comedy at the Haymarket Theatre.

A new play by Mr. Hubert Henry Davies, author of "Mrs. Gorringe's Necklace," has been produced at the Haymarket Theatre. "Cousin Kate" is likely to hold its place on the boards for some time. The plot is of the simplest. Amy Spencer is "all forlorn," for her Irish artist-lover, Heath Desmond, has left her on the eve of her wedding day, over a lover's quarrel, the reason being that Amy has Puritan tendencies, her *fiancé* has none. The Rev. James Bartlett comes in to console the bereaved girl, opines that "all will be for the best," and endeavours to prove it by courting Amy himself. At this point Cousin Kate, a woman of the world, arrives on the scene, puts a stop to the Rev. James's wooing, and makes Amy write a letter to recall Heath. When all seems smooth she once more goes to see to fires, etc., in the half-empty house where Amy is to live after her marriage. There she comes across a fascinating stranger to whom she had been much attracted when she met him recently in a railway carriage, and discovers the unknown to be her cousin's future husband. Heath Desmond makes the most of his chances, and in the process of boiling a kettle and listening to the "Three Bears" Cousin Kate finds her heart has been given to the Irishman. The act ends in a lovers' embrace, Amy walking in just in time to witness it. The third act straightens matters out, and Amy pairs off with the parson. The play is full of joyous fun and innocent gaiety, and is as refreshing as a sea-breeze. The principal parts are admirably acted.

### For Reading to the Sick.

### THE HALLOWING OF SUFFERING.

Sacrifice and Self-devotion hallow earth and fill the skies,
And the meanest Life is sacred—whence the highest may arise. *Houghton.*

And the holiest still are those
Who are farthest from repose,
And yet, onward, onward, press
To a loftier Godliness;—
Still becoming,—more than being,
Apprehending,—more than seeing,
Feeling, as from orb to orb
In their awful course they run,
How their souls new light absorb
From the Self-Existing One.
*Houghton.*

It is by the still strength of a holy character that we must leave the stamp of God upon the world.—*H. E. Manning.*

There are no circumstances so poor but that character may display itself and make itself therein. Strength of character lies not in demanding special circumstances, but in mastering and using any that may be given. Daily contact with our fellows forms our scene of action, and God blesses with a peculiar blessing the efforts to put to profit, not some self-selected occasion, but the actual conditions in which we find ourselves. . . .

The one enduring interest of human life is character, and our faith brightens our lives by unearthing character. . . . It proclaims that our "life is hid with Christ"; we are wholly free, nothing can hold us down; gifted with a refuge far above all accidents and risks, we have an indefeasible advantage over all earthly circumstances. "Hid with Christ, free with the freedom of the Son," nothing can thwart or terrify us; safe hid from the provoking of all men, no man can snatch us out of His Hand. . . .—*Canon Scott Holland.*

We are like to Him with whom there is no past or future, with whom a day is as a thousand years, and a thousand years as one day, when we do our work in the great present, leaving both past and future to Him to whom they are ever present, and fearing nothing, because He is in our future as much as He is in our past, as much as, and far more than, we can feel him to be in our present. Partakers thus of the divine nature, resting in that perfect All-in-all in whom our nature is eternal too, we walk without fear, full of hope and courage and strength to do His will, waiting for the endless good which He is always giving as fast as He can get us able to take it in.—*G. MacDonald.*

O hearts that faint
Beneath your burdens great, but make no plaint,
Lift up your eyes!
Somewhere beyond, the Life you give is found,—
Somewhere, we know, by God's own hand is crowned
Love's Sacrifice!
*Maria Drake.*

Live on, brave lives, chained to the narrow round
Of Duty!—Live! expend yourselves! and make
The orb of Being wheel onward steadfastly
Upon its path! The Lord of Life alone
Knows to what goal of Good:—work on! live on!
*L. Morris.*

### Notes and Queries.

FOR REGULATIONS SEE PAGE 136.

*Victorian Nurses wishing to come to England.*

(118) Three certificated Victorian nurses wishing to come to London, desire to know: (1) if they could join a hospital staff without having to go through the training, (2) and whether they would have any difficulty in joining a nursing home?—*J. R. J.*

(1) Yes, but the matrons of our large hospitals probably prefer nurses trained in English methods. (2) There would probably be little difficulty.

*South Africa.*

(119) 1. Having had experience in nursing, could I work my passage out to South Africa to a Johannesburg hospital to be trained? 2. Can you tell me the name of the society that is sending women out to South Africa?—*R. M. J. and M. G.*

1. No; you had better train in England first. 2. Write to the Hon. Secretary, the South African Expansion Emigration Committee, 47 Victoria Street, S.W.

*Pension.*

(120) A gentlewoman of 76, in feeble health, has suddenly become penniless. A relative, in poor circumstances, would try and keep her if a little pecuniary assistance could be obtained. Is there not some society for assisting poor gentlefolk?—*Sympathetic Nurse.*

The Distressed Gentlefolks' Aid Association, 75 Brook Green, London, W.; the National Benevolent Institution, 65 Southampton Row, Bloomsbury, S.W.; the United Kingdom Beneficent Association, 7 Arundel Street, Strand, W.C.; and the Universal Beneficent Society, 15 Soho Square, W.

*Dispensing.*

(121) I am anxious to become a dispenser; what had I better do?—*M. A.*

I should like to to take up dispensing sufficiently to enable me to do all that was necessary at a free dispensary. What would be the nearest college where I could be taught? what length of time would the course take? would Latin be essential? and what would be the approximate cost?—*C. M. L.*

Write to the Secretary, the Pharmaceutical Society, 17 Bloomsbury Square, W.C.

*Open-Air Sanatorium.*

(122) Is there any open-air sanatorium at Bournemouth which would receive a patient at 10s. a week?—*E. E.*

The National Sanatorium for Consumption and Diseases of the Chest, Bournemouth. Terms of admission by Governor's nomination and the payment of 7s. 6d. weekly. The cost of a single nomination is £5.

*Male Nurses.*

(123) Can you kindly tell me of a hospital, in the North of England preferred, where men are trained as nurses? I have had some experience in nursing during the late war.—*A. W. S.*

The National Hospital for the Paralysed and Epileptic, Queen's Square, Bloomsbury, W.C., is the only hospital in the United Kingdom which trains male nurses.

*Homes.*

(124) Can you tell me of any hospital or home, other than a workhouse infirmary, where a consumptive patient could be taken free of charge? It is a very sad case, there are no friends to help, and the patient is quite alone in the world.—*Sydenham.*

There are now a few Amalgamated County Sanatoria where the patient would be received if within the necessary districts. But consult your doctor as it is very important to know the condition of the patient before seeking a Home.

Will you kindly tell me the name of the "Home" at Buxton for the treatment of rheumatism, and also the steps necessary to gain admission?—*Nurse.*

Perhaps you refer to the "Devonshire Hospital and Buxton Bath Charity." Apply to the Secretary for particulars.

**Important Nursing Textbooks.**

"The Nursing Profession: How and where to Train." 2s. net; 2s. 4d. post free.

"A Handbook for Nurses." (New Edition). 5s. net; 5s. 4d. post free.

"The Human Body." 5s. post free.

"Ophthalmic Nursing." (New Edition). 3s. 6d. net; 3s. 10d. post free.

"Gynæcological Nursing." 1s. post free.

"Art of Feeding the Invalid." (Popular Edition). 1s. 6d. post free.

"Practical Hints on District Nursing." 1s. post free.

## Figures and Tables

**Fig. 1. f1:**